# Supplementary material for: Collective incentives reduce over-exploitation of social information in unconstrained human groups
Source: Nat Commun. 2024 Mar 27;15:2683. doi: 10.1038/s41467-024-47010-3 (PMC10973496; doi:10.1038/s41467-024-47010-3)
Supplement: Supplementary file 1 — Supplementary Information [file 41467_2024_47010_MOESM1_ESM.pdf]

Electronic Supplementary Material for

Collective incentives reduce over-exploitation of social information in unconstrained human groups

Dominik Deffner[1,2*], David Mezey[2,3], Benjamin Kahl[1], Alexander Schakowski[1], Pawel Romanczuk[2,3], Charley Wu[1,4,5] & Ralf Kurvers[1,2]

[1]Center for Adaptive Rationality, Max Planck Institute for Human Development, Berlin, Germany
[2]Science of Intelligence Excellence Cluster, Technical University Berlin, Berlin, Germany
[3]Institute for Theoretical Biology, Humboldt University Berlin, Berlin, Germany
[4]University of Tübingen, Tübingen, Germany
[5]Max Planck Institute for Biological Cybernetics, Tübingen, Germany
[*]deffner@mpib-berlin.mpg.de

Supplementary Movies
Supplementary Movie 1. Example of participants' first-person field of view during the experiment.
Supplementary Movie 2. Example video of in-game tutorial.
Supplementary Movie 3. Illustration of the visual field reconstruction.

Supplementary Figures
Supplementary Figure 1. Exploitation probabilities over time.
Supplementary Figure 2. Additional behavioral results.
Supplementary Figure 3. Behavioral results at the group level.
Supplementary Figure 4. Movement metrics and independent discoveries.
Supplementary Figure 5. Behavioral results for individual foragers.
Supplementary Figure 6. Movement metrics and independent discoveries for individual foragers.
Supplementary Figure 7. Exemplary time-series data for the Social Hidden Markov Decision model.
Supplementary Figure 8. State-dependent distributions from the Social Hidden Markov Decision model.
Supplementary Figure 9. Influence of average decision weights on collective foraging success.
Supplementary Figure 10. Temporal dynamics in state predictors using linear trends.
Supplementary Figure 11. Collective visual-spatial dynamics for up to 3-minute time lags.

Supplementary Tables
Supplementary Tables 1 & 3-7. Main behavioral results using frequentist generalized linear models.
Supplementary Table 2. Foraging success conditional on prior experience.

Supplementary Methods
Preregistration document (OSF link: https://osf.io/5r736/)

Supplementary Figures

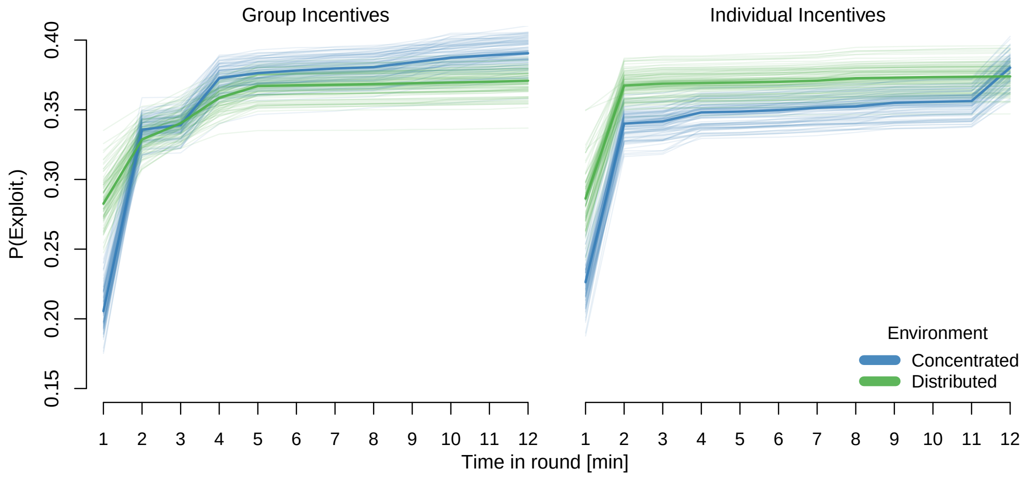

Supplementary Figure 1. Exploitation probabilities over time 100 random draws from the posterior distribution (transparent lines) as well as posterior means (solid lines) for the probability that players are successful (i.e., exploiting a patch) over time per incentive condition and environment. Results come from multilevel logistic regression with time in round as an ordered categorical (monotonic) predictor.

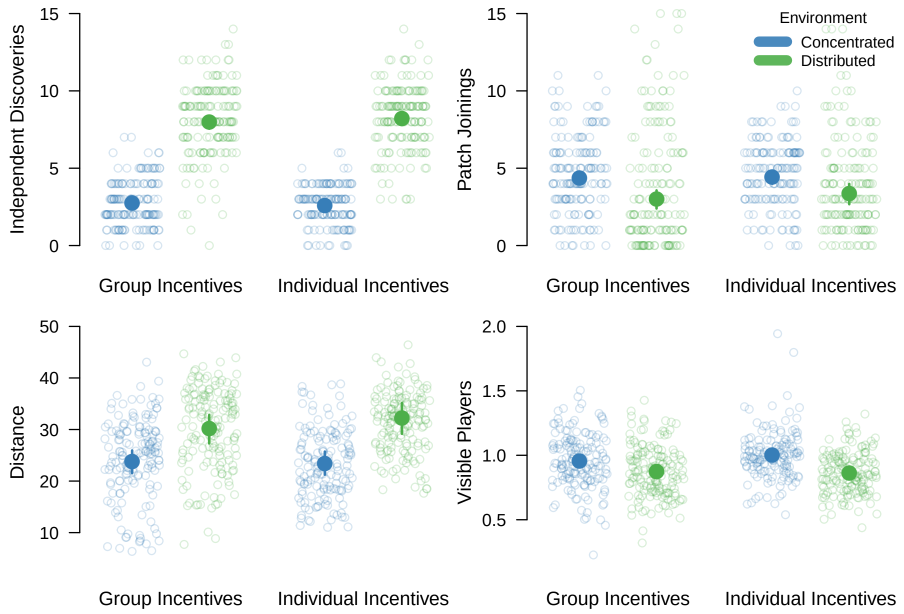

Supplementary Figure 2. Additional behavioral results. Number of independent patch discoveries and patch joinings, as well as distance (average distance to the other three players for times when focal player was not exploiting) and visibility (average number of players in field of view when focal player was not exploiting). Each circle represents data from one round per participant ($n = 160$), larger filled dots represent posterior means (as well as 90% HPDIs) from Bayesian multilevel models.

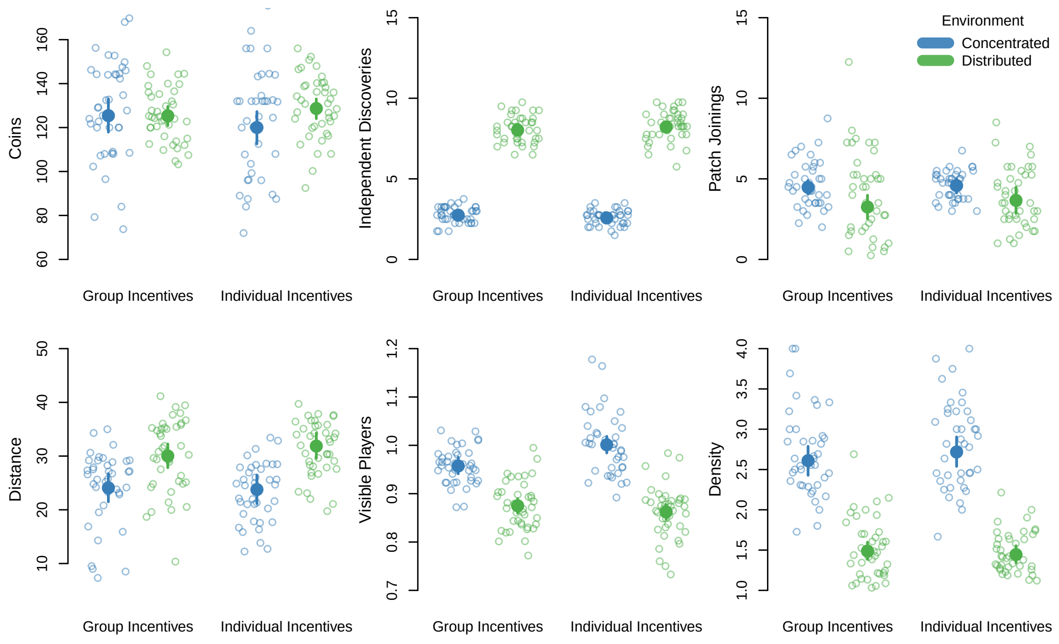

Supplementary Figure 3. Behavioral results at the group level. Average number of coins collected, independent patch discoveries, patch joinings, distance (average distance to the other three players for times when focal player was not exploiting), visibility (average number of players in field of view when focal player was not exploiting), and density (average number of foragers exploiting at discovered patches) per environment and incentive condition ($n = 40$). Each circle represents data from one round for each group of participants. Larger filled dots represent posterior means (as well as 90% HPDIs) from Bayesian multilevel models accounting for group-level variability.

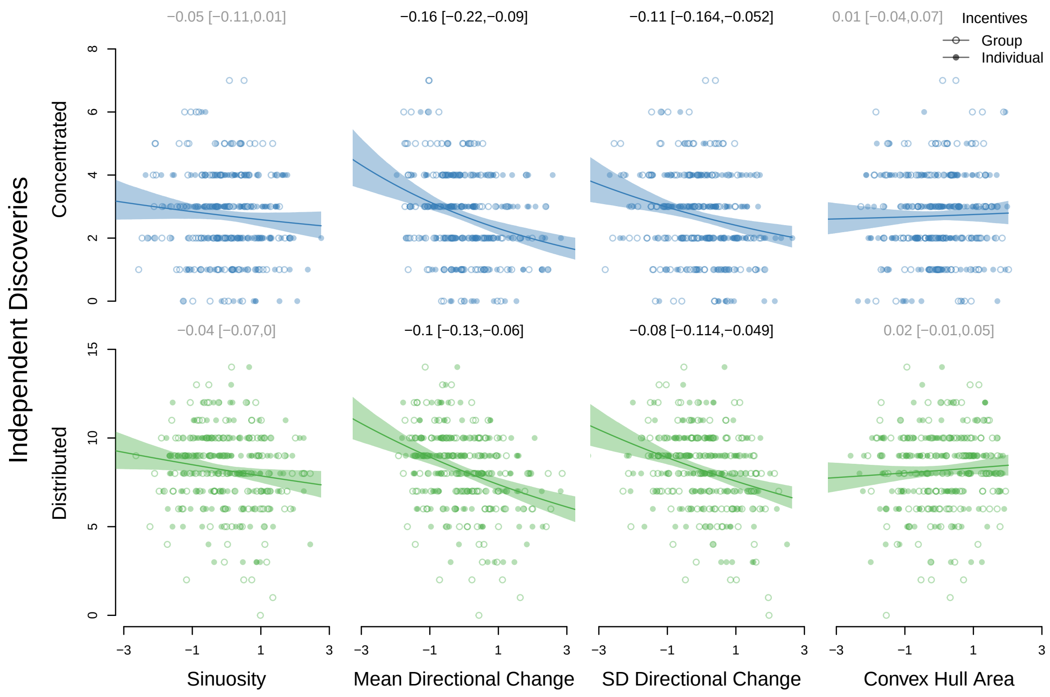

Supplementary Figure 4. Movement metrics and independent discoveries. Number of patches independently discovered in each round by individuals ($n = 160$) in concentrated and distributed environments as a function of different metrics describing each movement trajectory (using the trajr r package by McLean and Skowron Volponi, 2018): (1) Sinuosity index (Benhamou, 2004), (2) the mean of directional changes (measure of nonlinearity), (3) the standard deviation of directional changes (measure of irregularity) (Kitamura and Imafuku, 2015) as well as (4) the convex hull area (area of smallest convex set containing all locations a participant visited). Open circles represent participants in the “Group Incentives” condition, filled circles represent participants in the “Individual Incentives” condition. Lines and uncertainty intervals show the effect of each variable (reported above, transparent text if 90% HPDI overlaps 0) from Bayesian Poisson regressions accounting for baseline differences between conditions. All predictors were z-standardized before the analysis.

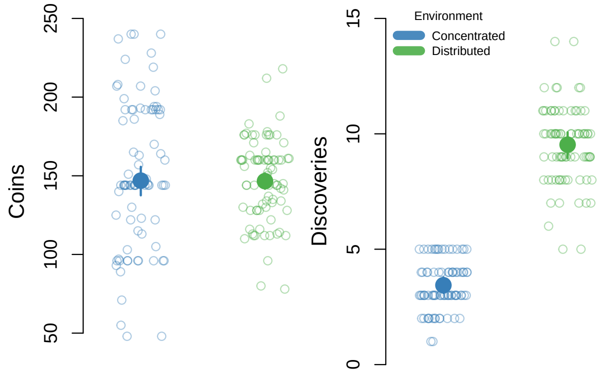

Supplementary Figure 5. Behavioral results for individual foragers. Number of coins collected and independent patch discoveries for different environments. Each transparent circle represents data from one round for one participant ($n = 40$). Larger filled dots represent posterior means (as well as 90% HPDIs) from Bayesian multilevel models.

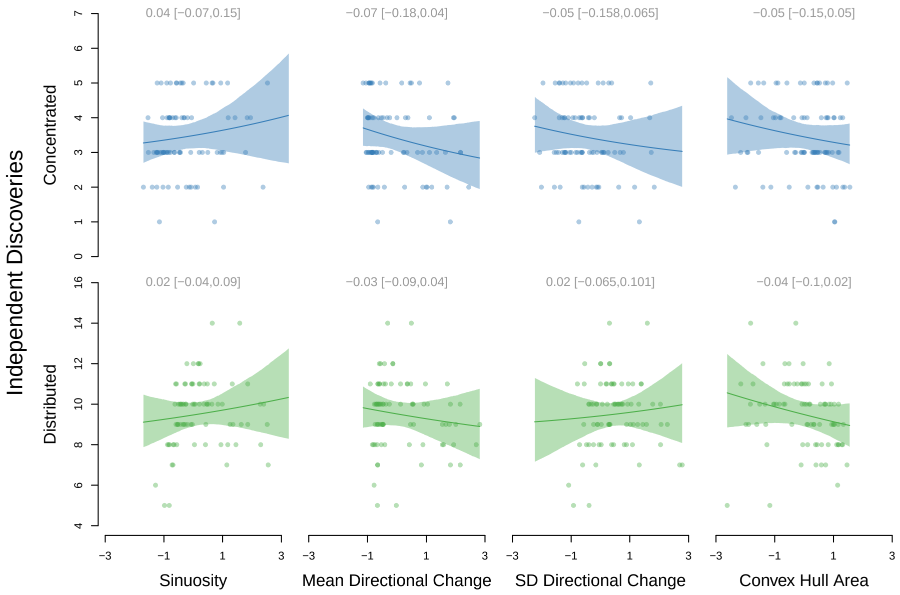

Supplementary Figure 6. Movement metrics and independent discoveries for individual foragers. Number of patches discovered in each round by individuals ($n = 40$) in concentrated and distributed environments as a function of different metrics describing each movement trajectory (using the trajr r package by McLean and Skowron Volponi, 2018): (1) Sinuosity index (Benhamou, 2004), (2) the mean of directional changes (measure of nonlinearity), (3) the standard deviation of directional changes (measure of irregularity) (Kitamura and Imafuku, 2015) as well as (4) the convex hull area (area of smallest convex set containing all locations a participant visited). Lines and uncertainty intervals show the effect of each variable (reported above, transparent text if 90% HPDI overlaps 0) from Bayesian Poisson regressions. All predictors were z-standardized before the analysis.

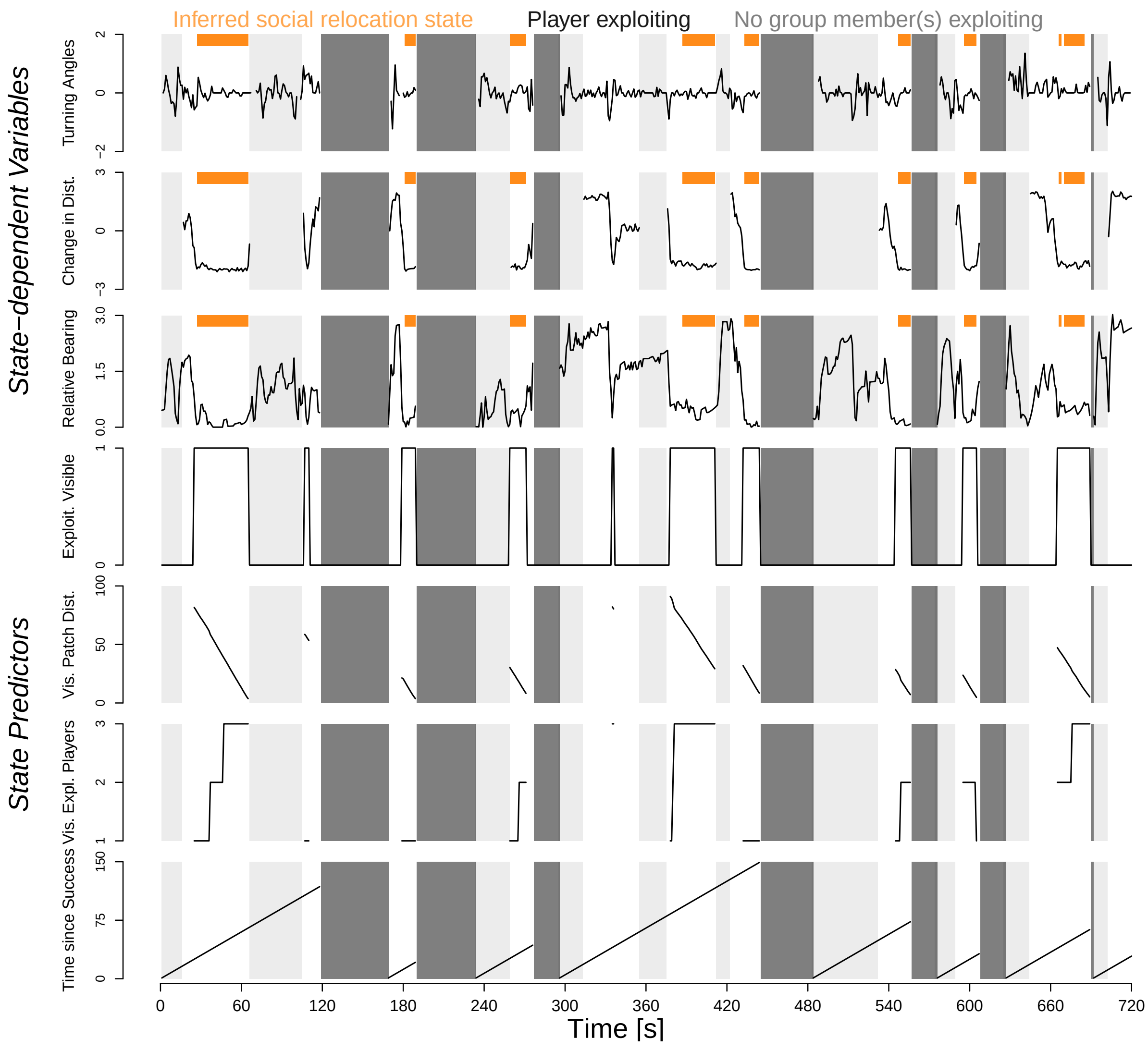

Supplementary Figure 7. Exemplary time-series data for the Social Hidden Markov Decision Model. *State-dependent Variables* used to identify latent behavioral states include (1) turning angles (change in movement direction between successive time points; in radians), (2) (smallest) change in distance to exploiting player(s) across time points and (3) relative bearing (smallest angle between orientation vector of player and locations of other players). *State Predictors* used to model latent transition probabilities between behavioral states include (1) a binary visibility indicator V ("Is any exploiting player in field of view?"), (2) the distance to the closest visible exploited patch D, (3) the number of other players extracting at the closest visible patch N as well as (4) the time since the last success (coin extraction) T. Trajectories show one data point per second, the same temporal resolution as used in the computational model. Exploitation times when a player was collecting coins at a patch are marked by dark grey areas. Lighter grey areas represent periods when no other player was collecting coins. In both cases, we know the state of the focal player (exploitation and individual exploration, respectively), so we only explicitly model periods when players potentially could use social information. Orange bars represent time periods identified by the Viterbi algorithm as social relocation.

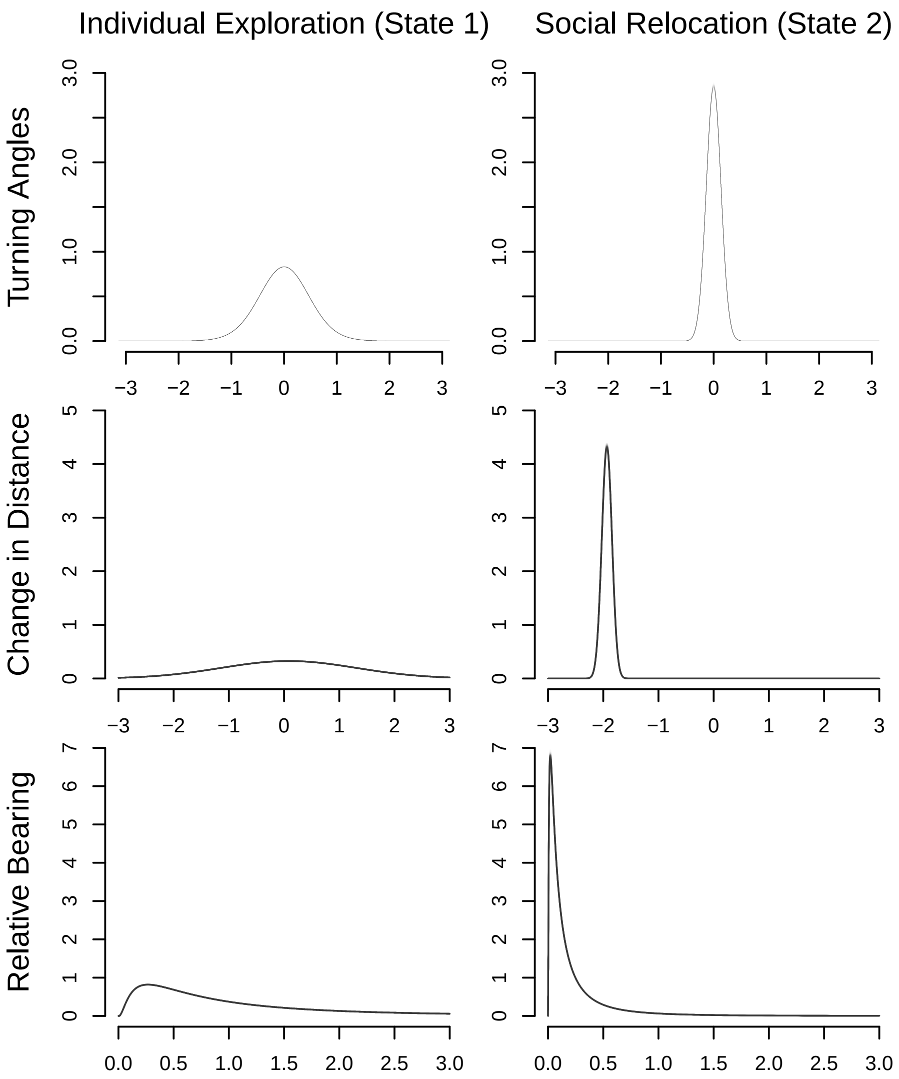

Supplementary Figure 8. State-dependent distributions from the Social Hidden Markov Decision Model. Von Mises distributions for turning angles (top), Gaussian distributions for change in distance to exploiting players (middle) and lognormal distributions for relative bearing (bottom) based on 100 random draws from the posterior for state 1 ("Individual Exploration") and state 2 ("Social Relacation"). Lines look almost identical due to the very narrow posterior parameter estimates defining those distributions, which are derived from almost 150k observations. Note the greater variability in behavior during individual exploration results in substantially wider distributions for all three variables .

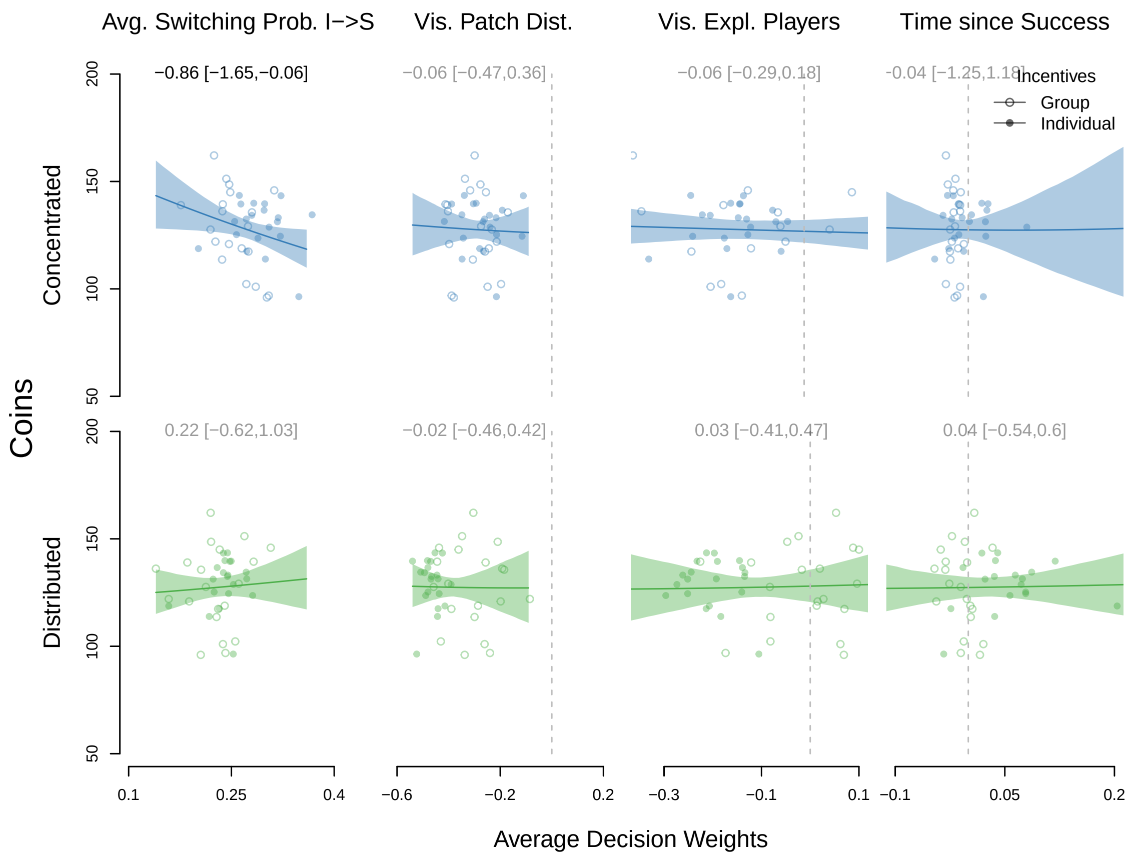

Supplementary Figure 9. Influence of average decision weights on collective foraging success. Success (average number of coins collected by each group; $n = 40$) in concentrated and distributed environments as a function of average individual-level weights derived from the Social Hidden Markov Decision Model. Open circles represent groups in the "Group Incentives" condition, filled circles represent groups in the "Individual Incentives" condition. Lines and uncertainty intervals show the effect of each decision weight (reported above, transparent text if 90% HPDI overlaps 0) from Bayesian log-normal regression models accounting for baseline success differences between conditions.

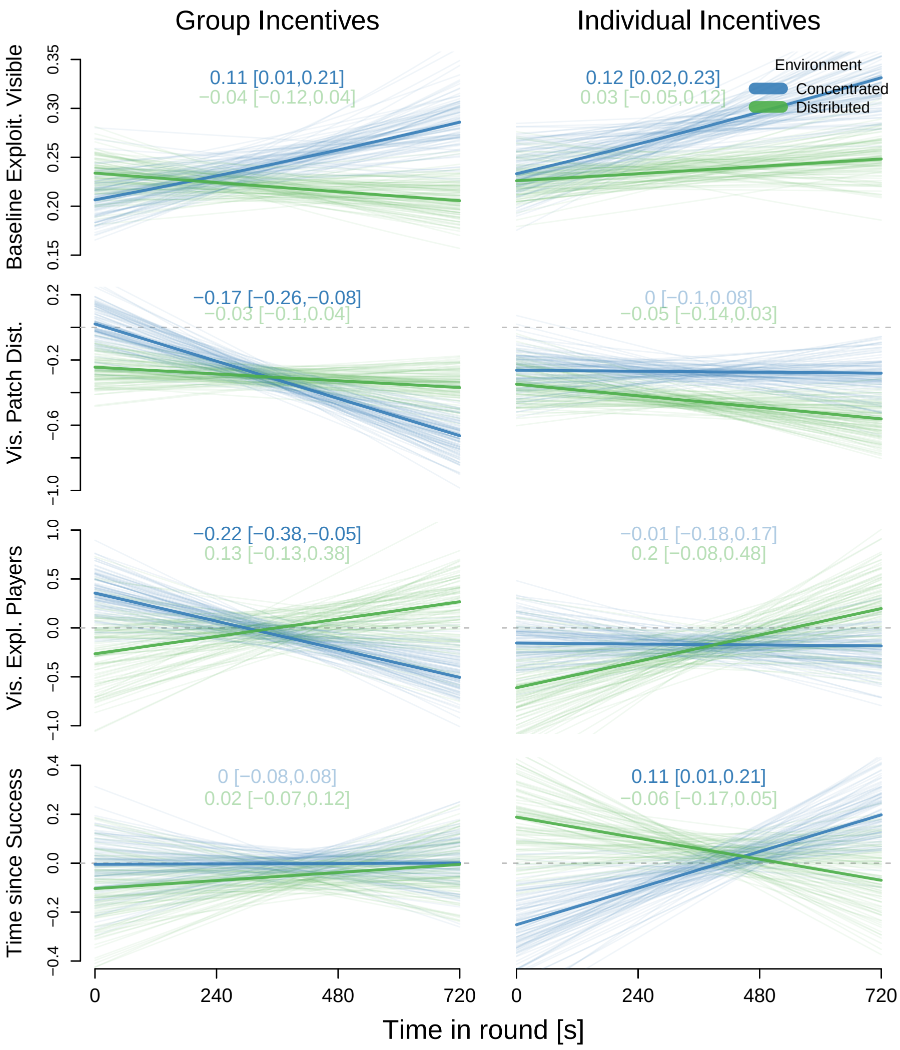

Supplementary Figure 10. Computational modelling results: Temporal dynamics in state predictors using linear trends. 100 random draws from the posterior distribution (transparent lines) as well as posterior means (solid lines) for the influence of different state predictors over the course of experimental rounds. The top row shows baseline switching probabilities across all situations in which participants observe (a) successful player(s), the following rows show deviations from this expectation on the logit scale.

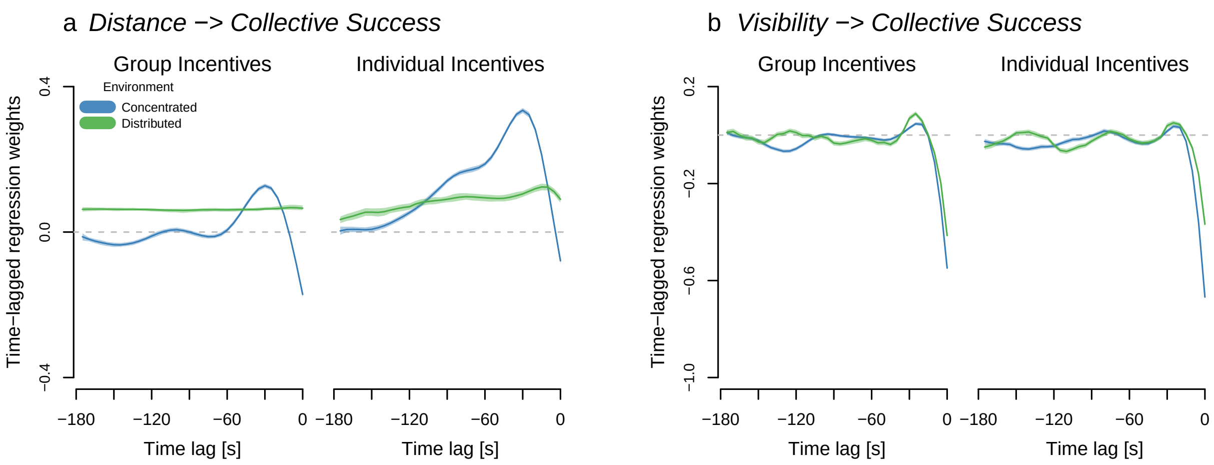

Supplementary Figure 11. Collective visual-spatial dynamics for up to 3-minute time lags. Time-lagged Gaussian-process regression weights (including 90% HPDIs) predicting collective foraging success (number of players exploiting a patch) based on (a) distance (average pairwise distance among players) and (b) visibility (number of visual connections among group members, ranging from 0, where no one is looking at others, and 12, where everyone is looking at everyone else) across different time intervals per incentive condition and environment.

Supplementary Tables

Supplementary Table 1. Frequentist analysis: Coins collected per incentive condition and environment (two-sided, Poisson likelihood, dummy-coded predictors)

	Estimate	SE	z	p-value
Intercept	4.01	0.01	394.21	$< 0.001^{***}$
Incentives	-0.04	0.01	-4.14	$< 0.001^{***}$
Environment	-0.01	0.01	-1.25	0.211
Incent. x Env.	0.07	0.02	4.95	$< 0.001^{***}$

Note: *p<0.05; **p<0.01; ***p<0.001

Supplementary Table 2. Expected number of coins collected (including 90% HPDIs) per environment (columns) depending on the environment in the previous round (rows). Results for group incentives are shown on the top, results for individual incentives are shown on the bottom.

Group Incentives

Previous	Concentrated	Distributed
First	125.0 [116.8, 133.5]	128.0 [122.6, 133.6]
Concentrated	124.7 [116.6, 133.2]	126.1 [121.6, 130.6]
Distributed	124.7 [116.9, 133.0]	118.6 [113.7, 123.6]

Individual Incentives

Previous	Concentrated	Distributed
First	121.8 [114.0, 130.0]	128.1 [122.8, 133.5]
Concentrated	109.8 [102.6, 117.2]	130.0 [125.4, 134.7]
Distributed	124.7 [116.9, 132.8]	125.5 [119.9, 130.2]

Supplementary Table 3. Frequentist analysis: Discoveries per incentive condition and environment (two-sided, Poisson likelihood, dummy-coded predictors)

	Estimate	SE	z	p-value
Intercept	1.06	0.07	15.24	$< 0.001^{***}$
Incentives	−0.06	0.07	−0.82	0.409
Environment	1.07	0.06	19.53	$< 0.001^{***}$
Incent. x Env.	0.08	0.08	1.04	0.298

Note: *p<0.05; **p<0.01; ***p<0.001

Supplementary Table 4. Frequentist analysis: Joinings per incentive condition and environment (two-sided, Poisson likelihood, dummy-coded predictors)

	Estimate	SE	z	p-value
Intercept	1.62	0.01	24.01	$< 0.001^{***}$
Incentives	−0.002	0.01	−0.03	0.975
Environment	−0.15	0.05	−2.69	0.007^{**}
Incent. x Env.	−0.04	0.07	−0.52	0.606

Note: *p<0.05; **p<0.01; ***p<0.001

Supplementary Table 5. Frequentist analysis: Distance to others per incentive condition and environment (two-sided, lognormal likelihood, dummy-coded predictors)

	Estimate	SE	z	p-value
Intercept	3.14	0.04	71.02	$< 0.001^{***}$
Incentives	−0.01	0.03	−0.37	0.713
Environment	0.24	0.03	6.96	$< 0.001^{***}$
Incent. x Env.	0.08	0.05	1.64	0.015^{*}

Note: *p<0.05; **p<0.01; ***p<0.001

Supplementary Table 6. Frequentist analysis: Visibility of others per incentive condition and environment (two-sided, Gaussian likelihood, dummy-coded predictors)

	Estimate	SE	z	p-value
Intercept	0.96	0.03	34.64	$< 0.001^{***}$
Incentives	0.05	0.02	2.07	0.043^{*}
Environment	−0.08	0.02	−3.85	$< 0.001^{***}$
Incent. x Env.	−0.06	0.03	−1.90	0.058

Note: *p<0.05; **p<0.01; ***p<0.001

Supplementary Table 7. Frequentist analysis: Scrounging rate per incentive condition and environment (two-sided, binomial likelihood, dummy-coded predictors)

	Estimate	SE	z	p-value
Intercept	0.49	0.09	4.99	$< 0.001^{***}$
Incentives	0.16	0.09	1.68	0.093
Environment	−1.59	0.07	−21.27	$< 0.001^{***}$
Incent. x Env.	−0.27	0.11	−2.51	0.012^{*}

Note: *p<0.05; **p<0.01; ***p<0.001

Supplementary Methods

Preregistration document (OSF link: https://osf.io/5r736/)

Social information use in virtual collective foraging

A Preregistration of Predictions and Analysis Plans

Dominik Deffner[1,2*], David Mezey[2,3], Benjamin Kahl[1], Charley Wu[1,4,5], Alexander Schakowski[1], Pawel Romanczuk[2,3] & Ralf Kurvers[1,2]

[1]Center for Adaptive Rationality, Max Planck Institute for Human Development, Berlin, Germany
[2]Science of Intelligence Excellence Cluster, Technical University Berlin, Berlin, Germany
[3]Institute for Theoretical Biology, Humboldt University Berlin, Berlin, Germany
[4]University of Tübingen, Tübingen, Germany
[5]Max Planck Institute for Biological Cybernetics, Tübingen, Germany
[*]deffner@mpib-berlin.mpg.de

SUMMARY

Here we outline a planned multiplayer immersive reality experiment to investigate the social decision making mechanisms underlying collective intelligence (and the failure thereof) in collective foraging. We (1) briefly describe the background, (2) explain the theoretical rationale and simulation results that inform our predictions, (3) introduce the experimental design using a novel collective behavior paradigm in immersive reality, (4) explain our predictions and (5) outline the analysis plan and a first state-recovery analysis using Social Hidden Markov Movement models and simulation.

1. Background

Intelligent behavior not only emerges from individual-level cognition, but also from collective computations occurring among groups of interacting agents. A fundamental challenge in collective problem solving in human, animal and robotic groups is the integration of personal and social information. Relying too heavily on personal information prevents the spread of useful information among group members, whereas relying too heavily on social information may reduce independent exploration of the environment and lead to maladaptive information cascades (Giraldeau et al., 2002; Rogers, 1988; Toyokawa et al., 2019). Here we investigate this key process by studying how human collectives dynamically balance personal and social information use across different environments to achieve collective intelligence. We aim to better understand the cognitive mechanisms, strategies and fundamental trade-offs underlying social learning and to identify the conditions that favor (or prevent) the emergence of collective intelligence in spatial search.

Collective foraging (i.e., searching for and extracting spatially distributed resources together) provides an ideal testbed to study the individual-level cognitive mechanisms and decision rules that underpin collective adaptation. All organisms need to efficiently identify and extract resources from their local environment and many animals not only forage alone but in groups with conspecifics (Giraldeau and Caraco, 2000). Unlike our closest primate relatives, humans have spread across the globe and now occupy a wider range than any other terrestrial animal (Henrich, 2017; Boyd and Silk, 2014; Mitani et al., 2012). Our species is able to survive and make a living in vastly different environments, ranging from tropical rainforests to the Arctic and from small-scale villages to megacities, and this immense adaptability is partly based on our ability to collectively find, extract, and process high-energy resources from our surroundings (Kaplan et al., 2000; Schuppli et al., 2012). Indeed, collective foraging involves several key ecological and social challenges that are considered important drivers of human brain-size evolution (González-Forero and Gardner, 2018). Such drivers include navigating and learning about complex and uncertain environments, cooperating with others to achieve shared goals, as well as competing against others to gain privileged access to resources (González-Forero and Gardner, 2018).

2. Theoretical rationale and simulation results

A rich body of theory on producer-scrounger games (e.g., Vickery et al., 1991; Barta et al., 1997; Giraldeau et al., 2002; Beauchamp, 2008; Kurvers et al., 2012; Giraldeau and Caraco, 2000) and information sharing (e.g., Clark and Mangel, 1984) has investigated the conditions that favor the evolution of social information use in collective foraging. In a spatially explicit model, Beauchamp (2008), for instance, has shown that a "scrounging" strategy, where agents look to join resource patches discovered by others instead of individually searching for resources ("producing"), increases in frequency (relative to "producers") when food patches are rich and the rate of patch encounter is low. When food patches are rich, scroungers can expect that a substantial amount of resource units will still be left once they arrive at a patch, increasing the value of social information. When food patches are rare and, therefore, hard to find, the expected benefits from searching independently are low, also favouring more scrounging. This model highlights the importance of taking realistic spatial constraints (e.g., travel times) into account. Including this key aspect of real biological systems generated qualitatively different predictions compared to previous models which assumed that scroungers instantaneously arrive at food patches, implying they would never arrive at a patch already depleted by others (e.g., Vickery et al., 1991).

Investigating foraging success on a collective level, models of collective search (e.g., Monk et al., 2018; Garg et al., 2022) have shown that social learning is favoured in rich and clustered environments, but also that collective performance depends on sufficient independent exploration of the resource landscape. In clustered environments, the behavior of others provides important cues about potential resource locations which other agents can exploit without engaging in costly individual search. If resources are more evenly distributed across the landscape, however, social information is of less use, as the observation of successful others will be less predictive of other available resources in the environment. Garg et al. (2022) further showed that selective use of social information (e.g., learn only from close-by agents) can mitigate the disadvantages of excessive social learning, especially in conditions where social information is prevalent (e.g., in large groups) or when individual exploration is limited. This shows that the strategic and selective use of social information is crucial for efficient collective foraging (see, e.g., Kendal et al., 2018, for literature on strategic social learning). Exploring the specific drivers of foraging strategies, Monk et al. (2018) have further identified that it is the "exploration difficulty" (search effort per unit of resource) and "exploitation potential" (value that can be extracted from patch after finding it) of a resource that controls the social behaviour of collective foragers. Irrespective of the specific details of a system, if resource units are hard to find through individual search but provide a large potential for exploitation once discovered, agents should be more likely to attend to the behavior or others.

Building on this work, we have recently developed a fully vision-based and spatially explicit mechanistic modeling framework of collectively foraging agents (see GitHub repo here for detailed information) that provides the theoretical foundation of the experimental work described below. Moving beyond previous work that modeled movement on a phenomenological level and omitted the underlying decision making processes and physical constraints (e.g., Garg et al., 2022; Monk et al., 2018; Barbier and Watson, 2016), we consider how collective intelligence in spatial search can arise mechanistically from the individual-level cognitive integration of personal and social cues.

Figure 1 shows two example snapshots of the agent-based simulation. Resource patches (grey circles) are initialized at random locations in the arena and can vary in the number of resource units they contain. This lets us directly manipulate the search difficulty and exploitation potential of resources in the environment (Monk et al., 2018). Each agent (colored circle) is characterized by a certain radius, a visual range (indicated by transparent green visual fields) as well as a velocity vector describing their orientation and absolute velocity.

Following Bartumeus et al. (2016), at each point in time, agents can be in one of three search states: "Exploit" (green), "Explore" (blue) or "Relocate" (purple). Exploitation and relocation are

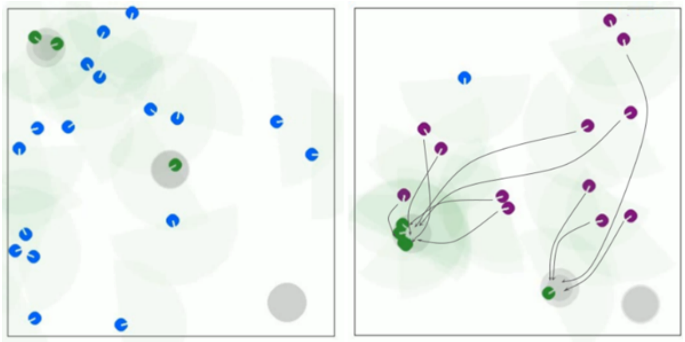

Figure 1. Example run of agent-based simulation model. Grey discs represent resource patches that are placed at random locations in the arena. Colored discs represent agents with white lines indicating current direction of movement and transparent green shades indicating their field of view. The colors represent the search state each agent is in: Exploiting agents are shown in green, relocating (i.e., scrounging) agents in purple and exploring agents in blue. The left plot shows a scenario of low "social excitability", the right plot shows a scenario of high "social excitability" (see text for more detailed explanation). The simulation is developed in the Python game engine *pygame* (Kelly, 2016).

associated with informed decision making, where agents either exploit a discovered resource patch or directedly move to another area resulting in relatively fast ballistic motion. In our collective foraging framework, relocation corresponds to socially induced "scrounging", where agents (aim to) join others who have located a new resource patch (Giraldeau and Caraco, 2000). In addition, there is a third, exploratory, state aimed at gathering more (personal or social) information.

Each state is characterized by a different set of movement parameters and we model the transitions between states and, thus, the movement of agents using stochastic differential equations describing the relative contribution of different (social and asocial) factors. Exploring agents continuously accumulate personal and social information based on the visual information they receive and switch to exploitation or relocation as soon as the respective decision thresholds are reached. Specifically, for social cues, we assume that agents respond to a binary visual projection field that accounts for the presence or absence of exploiting agents in their visual field. Conforming to the fundamental geometry of optics, this means that more agents and agents that are closer in space will exert proportionately stronger social influence. For personal cues, we simply assume that agents are able to perceive the resource quality at their present location. In the current implementation of the simulations, agents always start exploiting a resource patch once it is discovered and continue until it is empty. After exploitation, agents then switch back to the exploratory state.

To model cue integration and decision making, we use a leaky integrate and fire evidence accumulation process with two integrator variables that gradually accumulate personal or social information over time. Note that decision boundaries are fixed, so the speed of evidence accumulation alone determines behavioral responses to different cues. The weighing of social cues in the environment is controlled by a "social excitability" parameter ϵ that determines how quickly agents accumulate social information and, thereby, how strongly they respond to the behavior of others. For illustration, the left plot in Fig. 1 shows a scenario of low "social excitability" where agents ignore the social information provided by others and independently explore the environment instead of joining successful discoverers. The right panel in Fig. 1, on the other hand, shows a scenario of high "social excitability" where small amounts of social information in agents' visual fields are sufficient to make them switch to "social relocation" and move towards exploiting agents.

To investigate which levels of social learning improve collective performance in different kinds of environments, we conducted large-scale simulations systematically varying both the social excitability parameter ϵ and the distribution of resources in the environment. More specifically, we varied the number of patches, while keeping the total number of resource units constant across environments.

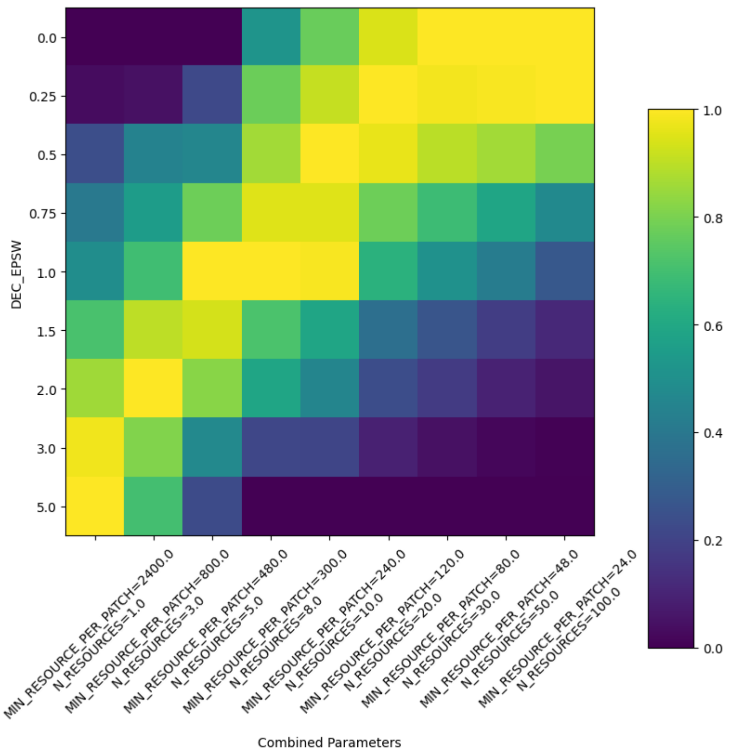

Figure 2. Results of the mechanistic, vision-based simulation model. Amounts of resources collected by all agents for different values of social excitability on the y-axis ($\epsilon = 0$ represents no social information use, $\epsilon = 5$ represents high levels of social information use) and patchiness of the environment on the x-axis (resources are concentrated in few but rich patches on the left and distributed across many but poor patches on the right). Lighter colors indicate better collective performance. Note that results are normalized for each type of environment (i.e., each column) such that 1 represents collective success for ϵ-value with highest efficiency and other values are proportions of this maximum collective efficiency.

Figure 2 shows collective search success (amount of resources collected by all agents; normalized for each column) for different values of social excitability (on the y-axis) and patchiness of the environment (on the x-axis). These simulation results show that groups of collectively foraging agents benefit from strong social learning in conditions where resource units are concentrated in a few, rich patches, while more individual exploration proves beneficial for the collective if resources are more evenly distributed across many patches in the environment.

Based on these and previous modeling results, we aim to test how groups of collectively foraging agents can dynamically balance individual exploration and the use of social information across different environments. We investigate how different resource distributions and incentive structures (individual vs. collective rewards) influence social learning strategies and how such strategies affect collective dynamics. We are particularly interested in which individual decision rules enable collective intelligence and when conflicts between individual and collective interests might prevent efficient collective behavior.

3. Paradigm and experimental design

In each group session of the experiment, four participants will control avatars in a virtual world (a "castle courtyard") and search for resources ("coins") that are hidden underground (see Fig. 3 for illustration). At the beginning of each round, a fixed number (depending on experimental condition, see below) of circular resource patches ("coin fields") with a radius of $r = 3$ "meters" are placed randomly across the arena (90x90 meters) ensuring that they do not overlap.

Over four rounds of twelve minutes each, participants use keyboard buttons to freely navigate through the virtual environment and detect resource patches with a metal detector. When individuals encounter a patch, they can start collecting coins by clicking on coin symbols that appear at different locations on the screen. New coins appear at a fixed interval of two seconds and stay on the screen until they are collected. This simple "mini game" is meant to ensure that participants stay engaged throughout the experiment without introducing additional sources of variation due to differential speed of extracting resources.

As soon as all coins of a patch are extracted, it disappears and a new patch containing the same number of coins is generated at a random location in the environment (while ensuring that it does not overlap with any existing patch or one of the participants). This means that the number of patches always stays constant within each round to reduce the effect of resource depletion over time.

Next to individual exploration of the environment, participants can also observe the behavior of others and decide to join players that have successfully discovered a resource patch. Avatars in the virtual environment will perform a digging movement using a shovel to indicate that they are currently extracting coins (see right avatar in Fig. 3). If multiple players simultaneously collect coins from the same patch, coins disappear at a rate that is proportional to the number of extracting players (2x as fast for two agents, 3x as fast for three,...). The virtual environment imposes a limited, first-person, field of view (76° in total) as well as realistic spatial constraints (maximum movement speed of 2 m/s) creating natural trade-offs between individual exploration of the environment and social learning (Wu et al., 2021; Bastien and Romanczuk, 2020).

The proposed experiment uses a simple 2x2 design. As a first (between-subjects) factor, we manipulate whether groups of participants are incentivized on the individual level ("competitive" condition) or on the group level ("cooperative" condition). In the competitive condition, participants are told to individually collect as many coins as possible to maximize their monetary reward at the end of the experiment. In the cooperative condition, in contrast, bonus payments are determined by the total number of coins collected by all participants and are distributed equally among team members. As a second (within-subjects) factor, we manipulate whether coins are concentrated in relatively few but rich patches ("concentrated" condition; 5 patches with 48 coins each) or are distributed among relatively many but poor patches ("distributed" condition; 15 patches with 16 coins each). Participants will experience each form of resource distribution for two out of the four rounds and all possible permutations of presentation order are realized for both incentive conditions across groups of participants. On top of 40 planned group sessions (20 in the competitive condition, 20 in the cooperative condition), approximately 40 additional participants will search for coins on their own providing an baseline control condition for comparison. Single participants will also experience the two resource distributions twice, and will be reimbursed based on their own performance.

At a sampling interval of 50Hz (50 times per second), for each player, we will record their X- and Y-coordinates, orientation vector, velocity, their current coin count, whether they are currently extracting at a resource patch and, finally, which other players are presently in their visual field. From this data, we can reconstruct full movement trajectories of all players, calculate their respective distances, relative orientations and available social information. In addition, we record all instances when a new patch was generated (including the coordinates of its center), when players discovered and extracted a coin from a patch and when a patch finally disappeared. For each event involving a player, we record the exact time stamp and the ID of the player that triggered the event.

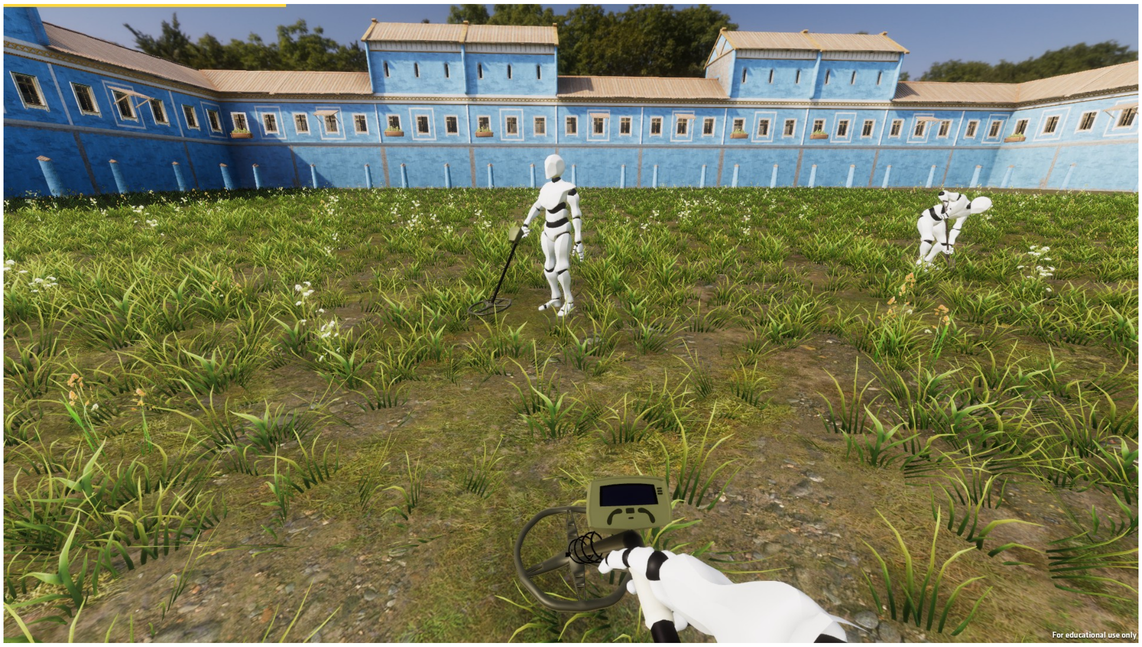

Figure 3. Example participant screen in collective foraging experiment.

4. Predictions

By analyzing movement patterns, visual information and social interactions, we will infer latent behavioral modes (individual exploration or social attraction) and social learning strategies (see section below for statistical analyses). This will hopefully allow us to investigate participant-specific degrees of social learning in different environments and inform us about which constellations of resource distributions, incentive structures and social decision rules favor (or prevent) the emergence of collective intelligence in collective spatial search. Specifically, we aim to test the following main set of predictions:

(1) Coins that are distributed among relatively few but rich patches are harder to find and provide a greater potential for exploitation. Therefore, we expect agents to rely more on social information in the "concentrated" resource condition compared to the "distributed" condition. This prediction follows directly from previous models of collective foraging (Barnard and Sibly, 1981; Beauchamp, 2008; Monk et al., 2018; Garg et al., 2022) as well as our own simulation results described above. Overall social learning will be quantified (1) through the relative frequency of patch joining events compared to independent discoveries as well as (2) the predicted proportions of a "social relocation" state in computational Hidden Markov models (see section on statistical analyses below).

(2) Theory on producer-scrounger dynamics (Giraldeau and Caraco, 2000; Giraldeau et al., 2002; Barta et al., 1997; Beauchamp, 2008; Kurvers et al., 2012) as well as the evolution of social learning (Rogers, 1988; Deffner and McElreath, 2022) has shown that social learning can be individually beneficial without improving collective performance or population fitness. As social learners capitalize on the discoveries of others, they can acquire adaptive behavior without engaging in potentially costly individual exploration. Moreover, as resources at each patch are limited, social "scrounging" lets players collect coins at the direct expense of "producers" that have discovered the patch. Based on these results, we expect overall more social learning in the competitive condition, where everyone aims to maximize their own payoff, compared to the cooperative condition, where participants aim to maximize the

success of the group. As before, social learning will be quantified through relative frequency of patch joining events and predicted proportion of time in "social relocation" state.

(3) Different amounts of social learning are optimal for individual and collective performance depending on the distribution of resources in the environment (Beauchamp, 2008; Garg et al., 2022). For instance, our simulation results described above show that high levels of social learning are beneficial for the collective if resources are concentrated in relatively few patches, while more individual exploration proves beneficial if resources are distributed across many patches. We expect that overall levels of social learning in cooperative groups, where individual and collective interests are aligned, will broadly conform to this pattern, such that individuals rely substantially more on social information in the "concentrated" condition compared to the "distributed" condition. In competitive conditions, however, where individual and collective interests diverge, we expect excessive levels of social learning that deviate from the collective optimum. This effect should be particularly visible in the "distributed" condition where collectives would benefit from high individual exploration while individuals might still benefit from large degrees of scrounging. In the "concentrated" condition, on the other hand, both individual and collective performance should benefit from high levels of social learning, so behavior in cooperative and competitive conditions should be comparatively more similar.

(4) Theoretical modeling has shown that for social learning to be adaptive, it generally needs to be strategic, e.g., selective with respect to which sources of information to follow (Kendal et al., 2018; Garg et al., 2022). In addition to adjusting the general reliance on social information, collective foragers might also mitigate the risk of maladaptive herding by strategically using different cues to decide which pieces of social information to follow. Garg et al. (2022), for instance, showed that incorporating social information conditional on the distance of other players can modulate the disadvantages of excessive social learning. Relatedly, players might also use the number of exploiting agents at a patch as a cue to predict how many coins might still be left when joining the patch. By "selective" social learning, we therefore mean that agents are more likely to decide to join nearer patches and patches where fewer others are currently exploiting. Based on theoretical results (Kendal et al., 2018; Garg et al., 2022), we expect agents to be more selective in their social information use in the "distributed" condition compared to the "concentrated" condition. If resource units are distributed among many poor patches, far-away patches and patches where multiple players are already exploiting will likely be depleted until a participant arrives there, whereas the rich patches in the "concentrated" condition might still contain a substantial number of coins even if they are relatively far away and multiple other agents are already exploiting there. Additionally, we also expect agents to be more selective in the "cooperative" compared to the "competitive condition", as cooperative agents should be driven to avoid maladaptive levels of social information use.

The "selectivity" of social learning will be measured through regression weights that quantify the impact of distance and player number on players' probability to switch into (or stay in) social relocation state (see section below).

Lastly, players might also adjust their social learning based on their own recent experiences and performance. Schlag (1998), for instance, proposed that an imitation rule termed "proportional imitation" (or more intuitively "copy when dissatisfied") might be a highly effective learning rule. In the context of our experiment, this means that players might become more likely to attend to others and try to join a patch as more time passes since their last coin collection.

(5) Finally, compared to solitary foragers, we expect that the performance of individuals in groups will depend on the incentive structure among them (cooperative vs. competitive).

While we expect individuals in cooperative groups to consistently perform better than solitary foragers, individuals in competitive groups might be characterized by higher variance in success and might even perform worse due to maladaptive herding depending on the level of social learning. The benefits of social foraging for cooperative groups should be particularly pronounced in the "concentrated" condition, where resource patches are hard to find on your own but provide a large potential for exploitation.

5. Statistical analyses

By analyzing movement patterns, visual information as well as social interactions, we aim to directly infer latent behavioral modes (individual exploration vs. social relocation) and learning strategies (influence of distance and number of observed others) across the different experimental conditions. For this purpose, we are currently developing a Social Hidden Markov movement modeling framework borrowing tools and concepts from animal movement ecology (Nathan et al., 2008; Leos-Barajas and Michelot, 2018; Auger-Méthé et al., 2021). A Hidden Markov model (HMM) is a doubly stochastic time series with an observation process and an underlying state process. The model uses separate data streams to assign each observation probabilistically to one of a fixed number of hidden states (in our case, individual exploration and social relocation). HMMs also allow us to infer transition probabilities between the search states as well as the (social and asocial) factors influencing such transitions. This way, we can, for instance, model how the number, distance and success of other agents in one's visual field affects the probability to stop exploring and move towards other group members.

A computational approach is necessary because observable metrics, such as patch joining events, are not direct indicators of underlying processes and several different processes can typically result in the same empirical pattern (Kandler and Powell, 2018; Barrett, 2019). Imagine, for instance, a player in the experiment has decided to use social information and move towards successful others but does not arrive at the patch before all coins are collected; or, more luckily, this player might independently discover a new patch on the way while they were relocating towards others. In both scenarios, we would not actually observe this player join a patch, although they decided to do so based on all available social cues. Conversely, we can also imagine scenarios where players "join" others accidentally, e.g., if another agent (who is currently not visible) has just discovered a patch and a player turns around and finds the same patch without ever actively using social information. Therefore, if we are interested in how different socio-ecological conditions influence social learning and how such strategic decision making affect individual and collective performance, we need to move beyond observable metrics and infer the latent strategies and choices that generate the observed movement.

We are currently implementing such as model using simulated data that match the structure of the actual experimental data. As we are still in the process of model development, the following details are subject to change. However, all modeling choices will be based on simulated data alone, not the real experimental data from human participants. At the moment, we consider the following data streams as indicators to differentiate between the two different behavioral modes:

(1) Turning angles (change in movement direction from one time step to the next): Social relocation is expected to be characterized by smaller turning angles compared to individual exploration as "scroungers" move consistently in the same direction.
(2) Step lengths (distance travelled): Relocation should result in larger step lengths. However, as there is a fixed upper limit for movement speed, this variable might not differ as much between states.
(3) Smallest absolute closed angle with other agents: For this metric, each time step, we calculate the absolute angles between the orientation vector of an agent (which direction they are facing) and the vector connecting the agent's center to the center of every other agent. We

then take the smallest of those values. This variable is close to 0 if an agent is moving directly towards another agent. Therefore, social relocation should be marked by smaller values compared to individual exploration.

(4) Change in distance to exploiting players: If agents are moving towards others to join them at a patch, the average distance to those who are currently collecting coins should be decreasing.

(5) Lastly, we may also consider the rate of change of those variables. Social relocation should be characterized by relatively persistent movement, marked by small *changes* in turning angles, smallest closed angles and change in distance.

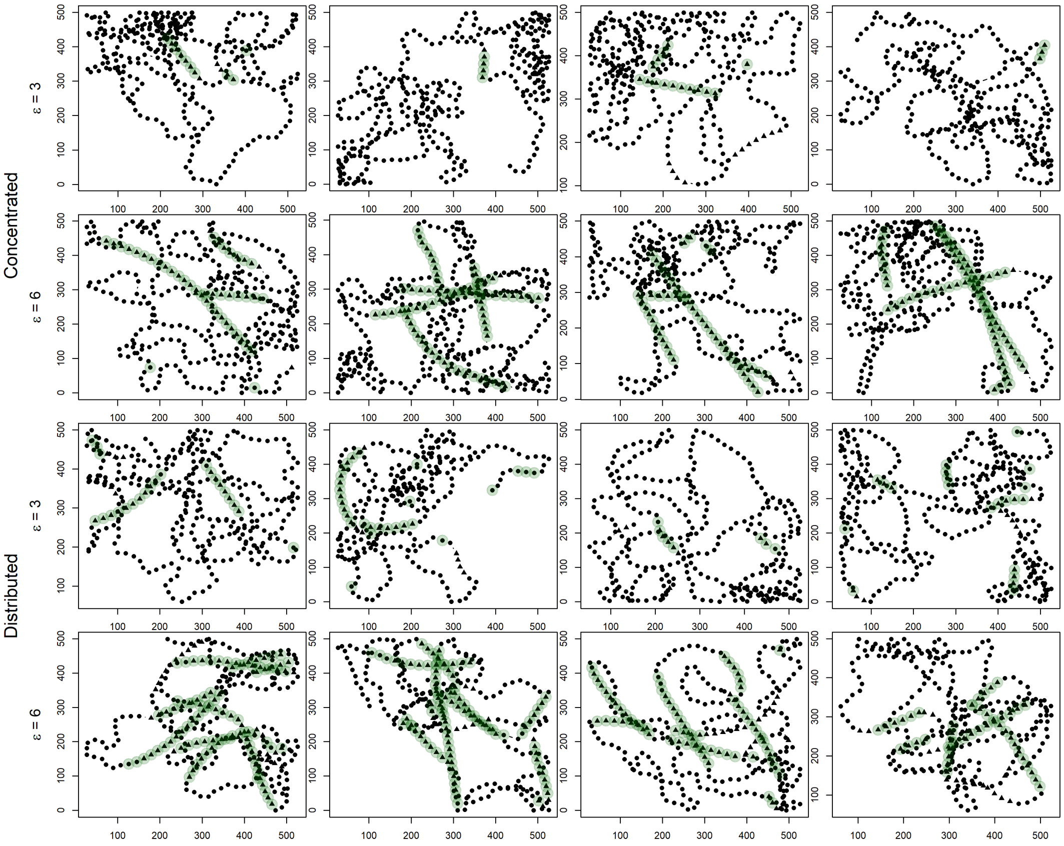

Figure 4. State recovery analysis of simulated collective foragers using Hidden Markov movement models.

Figure 4 shows the results of a first state-recovery analysis using the described Hidden Markov framework. We matched the parameter settings of the agent-based model described above to the design of the experiment and simulated 16 unique movement trajectories for different values of social excitability ($\epsilon = 3$ or $\epsilon = 6$) and both distributed and concentrated resource environments. Black dots represent time points when simulated agents were in exploratory state, black triangles represent times when agents were in social relocation state. Green transparent circles indicate reconstructed episodes of social information use (most likely state sequence for each movement trajectory was obtained from the posterior distribution through the Viterbi algorithm). Overall, the model is very well able to recover the "true" state of simulated agents. Note that unlike regular parameter recovery tests, the agents in the present case were simulated through a set of mechanistic stochastic differential equations instead of the probabilistic model used for inference, so we should not expect to see a perfect match. Nonetheless, the model correctly identified 98.8% of exploration episodes and

81.2% of social relocation episodes. This means the model is rather conservative in inferring episodes of scrounging/social information use. Inspecting Fig. 4, we can see that the model is especially likely to miss very short sequences of social information use and sequences where agents turned around during social relocation. This is expected as the present model only has access to movement data, but we hopefully will be able to improve inference using more refined state-dependent variables or including visibility information into state estimation.

Further, we will consider the following covariates to predict under which conditions agents switch from individual exploration to social relocation and vice versa. We only consider cues that are currently accessible to an agent.

(1) Distance to exploiting players in field of view (FoV).
(2) Number of exploiting players in FoV.
(3) Number of discovered patches in FoV.
(4) Times since last coin collected.

Using the Markov chains, we will quantify the proportion of time agents are expected to spend in each search mode across different experimental conditions. We can then compare those proportions with the optimal relocation times derived from our agent-based simulations. We will further compare the influence of social and asocial covariates on social learning for different resource distributions and incentive structures. Lastly, we will use the model estimates from human participants to simulate collective foragers in a larger range of different environments and determine under which conditions the observed social learning strategies might lead to intelligent group behavior or the collapse thereof.

References

Auger-Méthé, M., Newman, K., Cole, D., Empacher, F., Gryba, R., King, A. A., Leos-Barajas, V., Mills Flemming, J., Nielsen, A., Petris, G., et al. (2021). A guide to state–space modeling of ecological time series. *Ecological Monographs*.

Barbier, M. and Watson, J. R. (2016). The spatial dynamics of predators and the benefits and costs of sharing information. *PLoS computational biology*, 12(10):e1005147.

Barnard, C. J. and Sibly, R. M. (1981). Producers and scroungers: a general model and its application to captive flocks of house sparrows. *Animal behaviour*, 29(2):543–550.

Barrett, B. J. (2019). Equifinality in empirical studies of cultural transmission. *Behavioural processes*, 161:129–138.

Barta, Z., Flynn, R., and Giraldeau, L.-A. (1997). Geometry for a selfish foraging group: a genetic algorithm approach. *Proceedings of the Royal Society of London. Series B: Biological Sciences*, 264(1385):1233–1238.

Bartumeus, F., Campos, D., Ryu, W. S., Lloret-Cabot, R., Méndez, V., and Catalan, J. (2016). Foraging success under uncertainty: search tradeoffs and optimal space use. *Ecology letters*, 19(11):1299–1313.

Bastien, R. and Romanczuk, P. (2020). A model of collective behavior based purely on vision. *Science advances*, 6(6):eaay0792.

Beauchamp, G. (2008). A spatial model of producing and scrounging. *Animal Behaviour*, 76(6):1935–1942.

Boyd, R. and Silk, J. B. (2014). *How humans evolved*. WW Norton & Company.

Clark, C. W. and Mangel, M. (1984). Foraging and flocking strategies: information in an uncertain environment. *The American Naturalist*, 123(5):626–641.

Deffner, D. and McElreath, R. (2022). When does selection favor learning from the old? social learning in age-structured populations. *PloS one*, 17(4):e0267204.

Garg, K., Kello, C., and Smaldino, P. (2022). Individual exploration and selective social learning: Balancing exploration-exploitation trade-offs in collective foraging. *Journal of The Royal Society Interface*, 19.

Giraldeau, L.-A. and Caraco, T. (2000). *Social foraging theory*. Princeton University Press.

Giraldeau, L.-A., Valone, T. J., and Templeton, J. J. (2002). Potential disadvantages of using socially acquired information. *Philosophical Transactions of the Royal Society of London. Series B: Biological Sciences*, 357(1427):1559–1566.

González-Forero, M. and Gardner, A. (2018). Inference of ecological and social drivers of human brain-size evolution. *Nature*, 557(7706):554–557.

Henrich, J. (2017). *The secret of our success: How culture is driving human evolution, domesticating our species, and making us smarter*. Princeton University Press.

Kandler, A. and Powell, A. (2018). Generative inference for cultural evolution. *Philosophical Transactions of the Royal Society B: Biological Sciences*, 373(1743):20170056.

Kaplan, H., Hill, K., Lancaster, J., and Hurtado, A. M. (2000). A theory of human life history evolution: Diet, intelligence, and longevity. *Evolutionary Anthropology: Issues, News, and Reviews: Issues, News, and Reviews*, 9(4):156–185.

Kelly, S. (2016). Basic introduction to pygame. In *Python, PyGame and Raspberry Pi Game Development*, pages 59–65. Springer.

Kendal, R. L., Boogert, N. J., Rendell, L., Laland, K. N., Webster, M., and Jones, P. L. (2018). Social learning strategies: Bridge-building between fields. *Trends in cognitive sciences.*

Kurvers, R. H., Hamblin, S., and Giraldeau, L.-A. (2012). The effect of exploration on the use of producer-scrounger tactics. *PloS one*, 7(11):e49400.

Leos-Barajas, V. and Michelot, T. (2018). An introduction to animal movement modeling with hidden markov models using stan for bayesian inference. *arXiv preprint arXiv:1806.10639.*

Mitani, J. C., Call, J., Kappeler, P. M., Palombit, R. A., and Silk, J. B. (2012). *The evolution of primate societies.* University of Chicago Press.

Monk, C. T., Barbier, M., Romanczuk, P., Watson, J. R., Alós, J., Nakayama, S., Rubenstein, D. I., Levin, S. A., and Arlinghaus, R. (2018). How ecology shapes exploitation: a framework to predict the behavioural response of human and animal foragers along exploration–exploitation trade-offs. *Ecology letters*, 21(6):779–793.

Nathan, R., Getz, W. M., Revilla, E., Holyoak, M., Kadmon, R., Saltz, D., and Smouse, P. E. (2008). A movement ecology paradigm for unifying organismal movement research. *Proceedings of the National Academy of Sciences*, 105(49):19052–19059.

Rogers, A. R. (1988). Does biology constrain culture? *American Anthropologist*, 90(4):819–831.

Schlag, K. H. (1998). Why imitate, and if so, how?: A boundedly rational approach to multi-armed bandits. *Journal of economic theory*, 78(1):130–156.

Schuppli, C., Isler, K., and van Schaik, C. P. (2012). How to explain the unusually late age at skill competence among humans. *Journal of human evolution*, 63(6):843–850.

Toyokawa, W., Whalen, A., and Laland, K. N. (2019). Social learning strategies regulate the wisdom and madness of interactive crowds. *Nature Human Behaviour*, 3(2):183–193.

Vickery, W. L., Giraldeau, L.-A., Templeton, J. J., Kramer, D. L., and Chapman, C. A. (1991). Producers, scroungers, and group foraging. *The american naturalist*, 137(6):847–863.

Wu, C. M., Ho, M., Kahl, B., Leuker, C., Meder, B., and Kurvers, R. (2021). Specialization and selective social attention establishes the balance between individual and social learning. *bioRxiv.*

References

Benhamou, S. (2004). How to reliably estimate the tortuosity of an animal's path:: straightness, sinuosity, or fractal dimension? *Journal of theoretical biology*, 229(2):209–220.

Kitamura, T. and Imafuku, M. (2015). Behavioural mimicry in flight path of batesian intraspecific polymorphic butterfly papilio polytes. *Proceedings of the Royal Society B: Biological Sciences*, 282(1809):20150483.

McLean, D. J. and Skowron Volponi, M. A. (2018). trajr: An r package for characterisation of animal trajectories. *Ethology*, 124(6):440–448.
