# Supplementary material for: Collective incentives reduce over-exploitation of social information in unconstrained human groups
Source: Nat Commun. 2024 Mar 27;15:2683. doi: 10.1038/s41467-024-47010-3 (PMC10973496; doi:10.1038/s41467-024-47010-3)
Supplement: Supplementary file 3 — Description of Additional Supplementary Files [file 41467_2024_47010_MOESM3_ESM.pdf]

Description of Additional Supplementary Files

File Name: Supplementary Movie 1
Description: Example of participants' first-person field of view during the experiment. The movie shows the first-person perspective of a player first searching for a patch and then extracting coins. Coin images from PNGALL (https://www.pngall.com/usd-crypto-coin-png) licensed under CC BY-NC 4.0 (https://creativecommons.org/licenses/by-nc/4.0/deed.en).

File Name: Supplementary Movie 2
Description: Example video of in-game tutorial. This movie shows the full tutorial participants went through prior to the experiment. Coin images from PNGALL (https://www.pngall.com/usd-crypto-coin-png) licensed under CC BY-NC 4.0 (https://creativecommons.org/licenses/by-nc/4.0/deed.en).

File Name: Supplementary Movie 3
Description: Illustration of the visual field reconstruction. Red squares represent players with short green lines representing their current orientation. Lines are drawn between players if one player is in the visual field of another player.
