# Supplementary material for: Collective incentives reduce over-exploitation of social information in unconstrained human groups
Source: Nat Commun. 2024 Mar 27;15:2683. doi: 10.1038/s41467-024-47010-3 (PMC10973496; doi:10.1038/s41467-024-47010-3)
Supplement: Supplementary file 7 — Reporting Summary [file 41467_2024_47010_MOESM7_ESM.pdf]

nature portfolio

Corresponding author(s): Dr. Dominik Deffner

Last updated by author(s): Feb 9, 2024

Reporting Summary

Nature Portfolio wishes to improve the reproducibility of the work that we publish. This form provides structure for consistency and transparency in reporting. For further information on Nature Portfolio policies, see our Editorial Policies and the Editorial Policy Checklist.

Statistics

For all statistical analyses, confirm that the following items are present in the figure legend, table legend, main text, or Methods section.

n/a	Confirmed	
☐	☒	The exact sample size (*n*) for each experimental group/condition, given as a discrete number and unit of measurement
☐	☒	A statement on whether measurements were taken from distinct samples or whether the same sample was measured repeatedly
☐	☒	The statistical test(s) used AND whether they are one- or two-sided<br>*Only common tests should be described solely by name; describe more complex techniques in the Methods section.*
☐	☒	A description of all covariates tested
☐	☒	A description of any assumptions or corrections, such as tests of normality and adjustment for multiple comparisons
☐	☒	A full description of the statistical parameters including central tendency (e.g. means) or other basic estimates (e.g. regression coefficient) AND variation (e.g. standard deviation) or associated estimates of uncertainty (e.g. confidence intervals)
☐	☒	For null hypothesis testing, the test statistic (e.g. *F*, *t*, *r*) with confidence intervals, effect sizes, degrees of freedom and *P* value noted<br>*Give P values as exact values whenever suitable.*
☐	☒	For Bayesian analysis, information on the choice of priors and Markov chain Monte Carlo settings
☐	☒	For hierarchical and complex designs, identification of the appropriate level for tests and full reporting of outcomes
☒	☐	Estimates of effect sizes (e.g. Cohen's *d*, Pearson's *r*), indicating how they were calculated

Our web collection on statistics for biologists contains articles on many of the points above.

Software and code

Policy information about availability of computer code

Data collection	The experiment was implemented using the Unity game engine (version 2020.3.21, IL2CPP backend, built-in rendering pipeline, post-Processing Stack v2 3.1.1) using the Netcode for GameObjects library (version 1.0.0) with a Unity Transport layer.<br>The four instances for participants were connected to a local Windows Server running a Server Build of the experiment with a tick rate of 25Hz. Player movement was handled client-side. The Unity source code as well as built executives necessary to reproduce and run the experiment are stored on GitHub: https://github.com/DominikDeffner/VirtualCollectiveForaging, and have been archived within the Zenodo repository: https://doi.org/10.5281/zenodo.10650332.
Data analysis	The analysis code was written in R 4.0.3. Statistical models are fit using the Stan MCMC engine via the rstan (2.21.2), cmdstanr (0.5.3) and lme4 (1.1-34) packages. All analysis code is available on GitHub: https://github.com/DominikDeffner/VirtualCollectiveForaging, and has been archived within the Zenodo repository: https://doi.org/10.5281/zenodo.10650332.

For manuscripts utilizing custom algorithms or software that are central to the research but not yet described in published literature, software must be made available to editors and reviewers. We strongly encourage code deposition in a community repository (e.g. GitHub). See the Nature Portfolio guidelines for submitting code & software for further information.

Data

Policy information about availability of data

All manuscripts must include a data availability statement. This statement should provide the following information, where applicable:

- Accession codes, unique identifiers, or web links for publicly available datasets
- A description of any restrictions on data availability
- For clinical datasets or third party data, please ensure that the statement adheres to our policy

The full experimental data are available on GitHub: https://github.com/DominikDeffner/VirtualCollectiveForaging, and have been archived within the Zenodo repository: https://doi.org/10.5281/zenodo.10650332.

Research involving human participants, their data, or biological material

Policy information about studies with human participants or human data. See also policy information about sex, gender (identity/presentation), and sexual orientation and race, ethnicity and racism.

Reporting on sex and gender	A total of 200 individuals participated in the study. 123 self-identified as women, the rest identified as men (free text field was available for other gender identities). Our main theoretical questions did not concern effects of sex or gender and there are also no relevant theoretical predictions in our case on sex- or gender-based differences, so none of our analyses considered them separately. However, our open data includes information on self-reported gender, so interested researchers are able to investigate such differences. The terms "sex" and "gender" are not used in our manuscript.
Reporting on race, ethnicity, or other socially relevant groupings	We do not speak about race, ethnicity or class in our manuscript. However, we note in the Methods section that all participants "were proficient in German and most came from Western, educated, industrialized, rich, and democratic societies".
Population characteristics	Participants were on average 28.5 years old with a standard deviation of 6.4. We restricted the age of invited participants to 18-50, because we did not want to introduce additional sources of variation due to motor skills and experience with computers and games.
Recruitment	Participants were invited via email from the participant pool of the Max Planck Institute for Human Development. It is possible that participants with gaming experience were more likely to respond to the invitation. However, navigation in the game was very straightforward and there was an in-game tutorial to reduce the effects of prior experience as much as possible. In any case, we do not see how such differences might bias our overall conclusions on differences between conditions.
Ethics oversight	The study was approved by the Institutional Review Board of the Max Planck Institute for Human Development (number: A 2022-06) and participants signed an informed consent form prior to participation.

Note that full information on the approval of the study protocol must also be provided in the manuscript.

Field-specific reporting

Please select the one below that is the best fit for your research. If you are not sure, read the appropriate sections before making your selection.

- [ ] Life sciences
- [x] Behavioural & social sciences
- [ ] Ecological, evolutionary & environmental sciences

For a reference copy of the document with all sections, see nature.com/documents/nr-reporting-summary-flat.pdf

Behavioural & social sciences study design

All studies must disclose on these points even when the disclosure is negative.

Study description	Quantitative-experimental study using fine-grained visual and spatial data from participants interacting in an immersive environment.
Research sample	Convenience sample recruited from the institute participant pool (see above for age and gender information). The sample is not a typical student sample and is more diverse in terms of age and socio-economic background, but it is surely not representative of humanity as a whole. We do not know of any theory predicting cross-cultural variation in the dynamics we are interested in, but we would love to see more theoretical work on this as well as replications across diverse cultural contexts.
Sampling strategy	Participants were invited by randomly sending emails to members of the participant pool. We used simulations to conduct a state-recovery analysis using our Hidden Markov framework (https://osf.io/5r736/). We found that the length of the experiment was sufficient to estimate our key parameters of interest for each participant and our final sample size was determined by the available budget.
Data collection	Data were collected on networked computers in the behavioral lab of the Max Planck Institute for Human Development in Berlin by a professional lab manager who was overseeing data collection from a room next to the experimental room. The experimenter was blinded to the aim of the study and the hypotheses. Participants in groups were seated in the same room; opaque desk divider panels ensured that they could not observe each other's screen and mice with silent buttons prevented them from hearing when others clicked.
Timing	July-September 2022
Data exclusions	No participants were excluded. There were two group rounds (out of 160) with gaps in the time-series due to technical error. These data were excluded for all analyses requiring complete movement trajectories (e.g., Hidden Markov models and behavioral scrounging analyses.
Non-participation	No participants dropped out or declined participation.
Randomization	Participants were randomly allocated to experimental incentive conditions (group vs. individual). Resource environments were varied within-subjects and the presentation order was counterbalanced such that all combinations were realized across groups.

Reporting for specific materials, systems and methods

We require information from authors about some types of materials, experimental systems and methods used in many studies. Here, indicate whether each material, system or method listed is relevant to your study. If you are not sure if a list item applies to your research, read the appropriate section before selecting a response.

Materials & experimental systems

n/a	Involved in the study
☒	☐ Antibodies
☒	☐ Eukaryotic cell lines
☒	☐ Palaeontology and archaeology
☒	☐ Animals and other organisms
☒	☐ Clinical data
☒	☐ Dual use research of concern
☒	☐ Plants

Methods

n/a	Involved in the study
☒	☐ ChIP-seq
☒	☐ Flow cytometry
☒	☐ MRI-based neuroimaging
